# Hepatitis E Virus in Pork Food Chain, United Kingdom, 2009–2010

**DOI:** 10.3201/eid1808.111647

**Published:** 2012-08

**Authors:** Alessandra Berto, Francesca Martelli, Sylvia Grierson, Malcolm Banks

**Affiliations:** Animal Health and Veterinary Laboratories Agency, Weybridge, UK

**Keywords:** Hepatitis E, hepatitis E virus, foodborne disease, pork, food chain, PCR, viruses, United Kingdom

## Abstract

We investigated contamination by hepatitis E virus (HEV) in the pork production chain in the United Kingdom. We detected HEV in pig liver samples in a slaughterhouse, in surface samples from a processing plant, and in pork sausages and surface samples at point of sale. Our findings provide evidence for possible foodborne transmission of HEV during pork production.

During the past 10 years, hepatitis E virus (HEV) infection acquired in industrialized regions worldwide in the apparent absence of contaminated drinking water for fecal–oral transmission has been reported (*1*). In Japan, foodborne transmission of HEV through ingestion of contaminated Sika deer, wild boar, and pig meat has been demonstrated (*2*) and, in France, after ingestion of pig liver sausage (*3*). Detection of HEV in pig liver sold in retail locations has been reported from Japan, the United States, and the Netherlands in 1.9%, 14.0%, and 6.5%, respectively (*4**,**5*). PCR indicated that 1 (1.3%) of 76 pig livers collected at retail outlets in southwestern England was positive for HEV (*6*).

The European Commission Framework Program 7 project, Integrated Monitoring and Control of Foodborne Viruses in European Food Supply Chains, aimed to gather data on virus contamination of food and environmental sources for quantitative viral risk assessment and development of virus-specific guidance for food supply chain operators. The UK Animal Health and Veterinary Laboratories Agency investigated the pork food chain for HEV from slaughterhouse to point of sale. We investigated fecal contamination of pork and work surfaces during this study.

## The Study

During September 2009–October 2010, we collected samples from slaughtered pigs, human hands, and the environment at perceived critical points for virus contamination in a pig slaughterhouse, a processing plant, and 3 points of retail sale. Food safety fact-finding visits were made to the premises during which, through direct observations of conditions and practices, more points were identified where contamination with viruses possibly could occur and from where ad hoc surface samples were collected by using sterile gauze swabs and placed in in phosphate-buffered saline plus antimicrobial drugs.

We tested all samples collected by using real-time reverse transcription PCR (rRT-PCR) (*7*) for HEV. In addition, samples were tested for porcine adenovirus (PAdV) and human adenovirus by PCR as indicators of porcine and human fecal contamination, respectively. Nucleic acid extraction and rRT-PCR were performed according to standardized Integrated Monitoring and Control of Foodborne Viruses in European Food Supply Chains protocols. An extraction control virus, murine norovirus (MNoV), was placed in all samples before the lysis step of the extraction (*8*) to demonstrate the extraction of amplifiable nucleic acid. We performed all rRT-PCRs with an internal amplification control (*8*).

At the slaughterhouse, 40 carcasses were selected. Ten carcasses were randomly selected from each of 4 groups of pigs slaughtered that day; each group corresponded to a different farm of origin. From each carcass, the visceral pack was removed and 2–3 g of liver and 8–10 g of feces were collected. Also, 10 swab samples were collected from the handlers and environment (Table 1).

**Table 1:** Surfaces sampled to investigate hepatitis E virus in the pork food chain, United Kingdom, 2009–2010*

Slaughterhouse	Processing/cutting plant	Point of sale
Surface swab		
Bar under operator inspecting livers	Bench on which meat is sold	Chopping board
Floor under carcasses in clean area	Box in which cuts are collected	Slicer
Floor under which livers are hung	Door handle	Cold-room door handle
Boxes in which livers are collected before freezing and sale	Hook	Sausage maker
Knife used immediately after scraping	General purpose knife	General purpose knife
Knife used on livers	Point	NT
	Saw	Toilet
	Scale	Sink
Swab, handlers’ skin		
Hand 1	Hand 1	Hand 1
Hand 2	Hand 2	Hand 2
Hand 3	NT	NT
Hand 4	NT	NT

*NT, sample not taken.

At the processing plant, 10, 18, and 14 carcasses from 3 slaughterhouses, representing 12 farms of origin, were randomly selected. The set of 14 carcasses came from the slaughterhouse we had visited, but for logistical reasons the carcasses were not the same as those that we sampled at the slaughterhouse. We collected 5 g of muscle from the ventral abdomen of each carcass. The extraction control MNoV was not detected in 2 of the samples, which we therefore excluded from the study, leaving 40 samples. Also, 10 surface swab samples were collected (Table 1).

At points of retail sale, 75 sausages were collected in 11 batches from 3 locations representing 2 types of retail outlet (2 UK supermarket chains and 1 butcher). Sausages were collected on different days to ensure that they were from different batches of pigs. MNoV was not detected in 12 of the samples, leaving 63 sausages for investigation. Eight surface swab samples were collected at point of sale (Table 1).

HEV RNA was detected at each of 3 sites in the pork food supply chain (Table 2). At the slaughterhouse, 5 (13%) of 40 fecal samples, 1 (3%) of 40 livers, and 1 (25%) of 4 swabs of workers’ hands were HEV positive. At the processing plant, each of 40 pig muscle samples was negative for HEV, and a surface swab from a metal point used to hook the carcasses was HEV positive. At points of sale, 6 (10%) of 63 sausages and 2 (25%) of 8 surface samples (knife and slicer swabs) were HEV positive. Five of the 6 positive sausages were in 1 of the 11 batches collected. Control results showed no evidence of cross-contamination in the laboratory.

**Table 2:** Prevalence of PAdV, HEV, and HAdV in the pork food chain, 2009–2010*

Point in chain	Sample type	No. PAdV positive/ no. collected (%)	No. HEV positive/ no. collected (%)	No. HAdV positive/ no. collected
Slaughterhouse	Feces	39/40 (98)	5/40 (13)	NT
	Liver	6/40 (15)	1/40 (3)	NT
	Surface swab	4/10 (40)	1/10 (10)	0/10
Processing plant	Muscle	0/40	0/40	NT
	Surface swab	0/10	1/10 (10)	0/10
Point of sale	Sausage	0/63	6/63 (10)	NT
	Surface swab	1/8 (13)	2/8 (25)	0/8

*PAdV, porcine adenovirus; HEV, hepatitis E virus; HAdV, human adenovirus; NT, sample not taken.

The indicator of pig fecal contamination, PAdV, was detected at 2 of 3 sites (Table 2). Of 40 fecal samples, 39 (98%) from the slaughterhouse were PAdV positive , as were 6 (15%) of 40 livers and 4 (40%) of 10 swab samples (knife swab immediately after evisceration, 2 hand swabs, and floor swab from area under which pigs were hung). At the processing plant, PAdV was not detected in any of the 40 pig muscle samples or the 10 swab samples tested. At points of sale, PAdV was not detected in any of the 63 sausages tested, but 1 (13%) of the 8 swab samples from the door handle of the cold room was PAdV positive. This finding could have resulted from transfer from a contaminated pig carcass, but the in-test controls and method of sampling exclude this contamination as a source of the HEV in the sausage meat. Human adenovirus was not detected in any samples from any of the locations.

## Conclusions

In industrialized regions, although the incidence of clinical hepatitis E in humans is low, the seroprevalence of antibodies against HEV is relatively high (*9*), indicating a high proportion of subclinical disease and/or underdiagnosis (*10*). A small proportion of this exposure to HEV is likely to result from travel to regions to which the virus is endemic or migration from such regions (*11*). However, a substantial level of exposure to HEV seems to have an indigenous source.

Pork products are eaten in several industrialized regions, including the United Kingdom (*2**,**12**,**13*). A recent cluster of illnesses in southern France was associated with ingestion of *figatelli*, a pig liver sausage mainly eaten raw (*3*). Foodborne illness has been associated with ingestion of pig liver and pig muscle tissue (*14**,**15*). Our study showed that in the United Kingdom, pork products with high-volume nationwide consumption might be contaminated with HEV. We chose sausages as the type of point-of-sale pork product investigated for HEV because they are widely eaten throughout the United Kingdom (>212,746 tons in Great Britain during February 2011–February 2012, BPEX, Agriculture and Horticulture Development Board, UK), unlike pig liver, and a 10% HEV detection rate in pork sausages at point of sale could be a cause for concern.

In terms of the potential for virus transmission, the surface swabs provided evidence that PAdV and HEV contamination occurs in the slaughterhouse and at point of sale. From the processing plant, HEV was detected on 1 surface swab. The 98% positive rate recorded for pig feces with the PAdV indicator validates this approach to detecting fecal contamination of porcine origin. No evidence of human fecal contamination was detected at any point in the chain, indicating that personal hygiene standards were high and that the HEV detected was unlikely to have come from human contamination of the samples.

Because the numbers of samples tested for viral contamination in this study were relatively small, these results should be taken as indicators only. For greater confidence in the results, we recommend a larger study. In addition, we will assess the viability and genotype of HEV by further work of in vitro culture and nucleotide sequencing, respectively.
